# A case of anaphylaxis to peppermint

**DOI:** 10.1186/1710-1492-10-6

**Published:** 2014-01-28

**Authors:** Roian Bayat, Rozita Borici-Mazi

**Affiliations:** 1 Division of Respiratory Medicine Department of Medicine, Queen’s University, Kingston, ON, Canada; 2 Department of Medicine and Pediatrics Division of Allergy and Immunology, Queen’s University, 166 Brock Street, Kingston, ON K7L 5G2, Canada

**Keywords:** Anaphylaxis, Peppermint, Menthol, IgE mediated

## Abstract

**Background:**

Anaphylaxis, a form of IgE mediated hypersensitivity, arises when mast cells and possibly basophils are provoked to secrete mediators with potent vasoactive and smooth muscle contractile activities that evoke a systemic response. We report a case of IgE mediated anaphylaxis to peppermint (Mentha piperita) in a male shortly after sucking on a candy.

**Case presentation:**

A 69 year old male developed sudden onset of lip and tongue swelling, throat tightness and shortness of breath within five minutes of sucking on a peppermint candy. He denied lightheadedness, weakness, nausea, vomiting, or urticaria. He took 25 mg of diphenhydramine, but his symptoms progressed to onset of cough, wheeze and difficulty with talking and swallowing. He was rushed to the nearest emergency department, where he was treated with intramuscular epinephrine, antihistamines and steroids. On history, he reported recent onset of mouth itchiness and mild tongue and lip swelling after using Colgate peppermint toothpaste. He denied previous history of asthma, allergic rhinitis, food or drug allergies. His past medical history was remarkable for hypercholesterolemia, gastroesophageal reflux and gout. He was on simvastatin, omeprazole, aspirin, and was carrying a self-injectable epinephrine device. He moved to current residence three years ago and cultivated mint plants in his backyard. He admitted to develop nasal congestion, cough and wheeze when gardening. Physical examination was unremarkable apart from slightly swollen pale inferior turbinates. Skin prick test (SPT) was strongly positive to a slurry of peppermint candy and fresh peppermint leaf, with appropriate controls. Same tests performed on five healthy volunteers yielded negative results. Skin testing to common inhalants including molds and main allergenic foods was positive to dust mites. Strict avoidance of mint containing items was advised. Upon reassessment, he had removed mint plants from his garden which led to resolution of symptoms when gardening.

**Conclusion:**

IgE mediated anaphylaxis to peppermint is rare. This case demonstrates a systemic reaction to a commonly consumed item, incapable of triggering anaphylaxis in the far majority of the population, yet causing a severe episode for our patient.

## Background

Food allergy appears to be increasing in prevalence and is estimated to affect >2% of population [1]. More than 170 foods have been identified as being potentially allergenic, however a minority of these foods cause the majority of reactions [2]. We report a case of IgE mediated anaphylaxis to peppermint (Mentha piperita) in a male shortly after sucking on a candy.

## Case presentation

**Figure 1:**
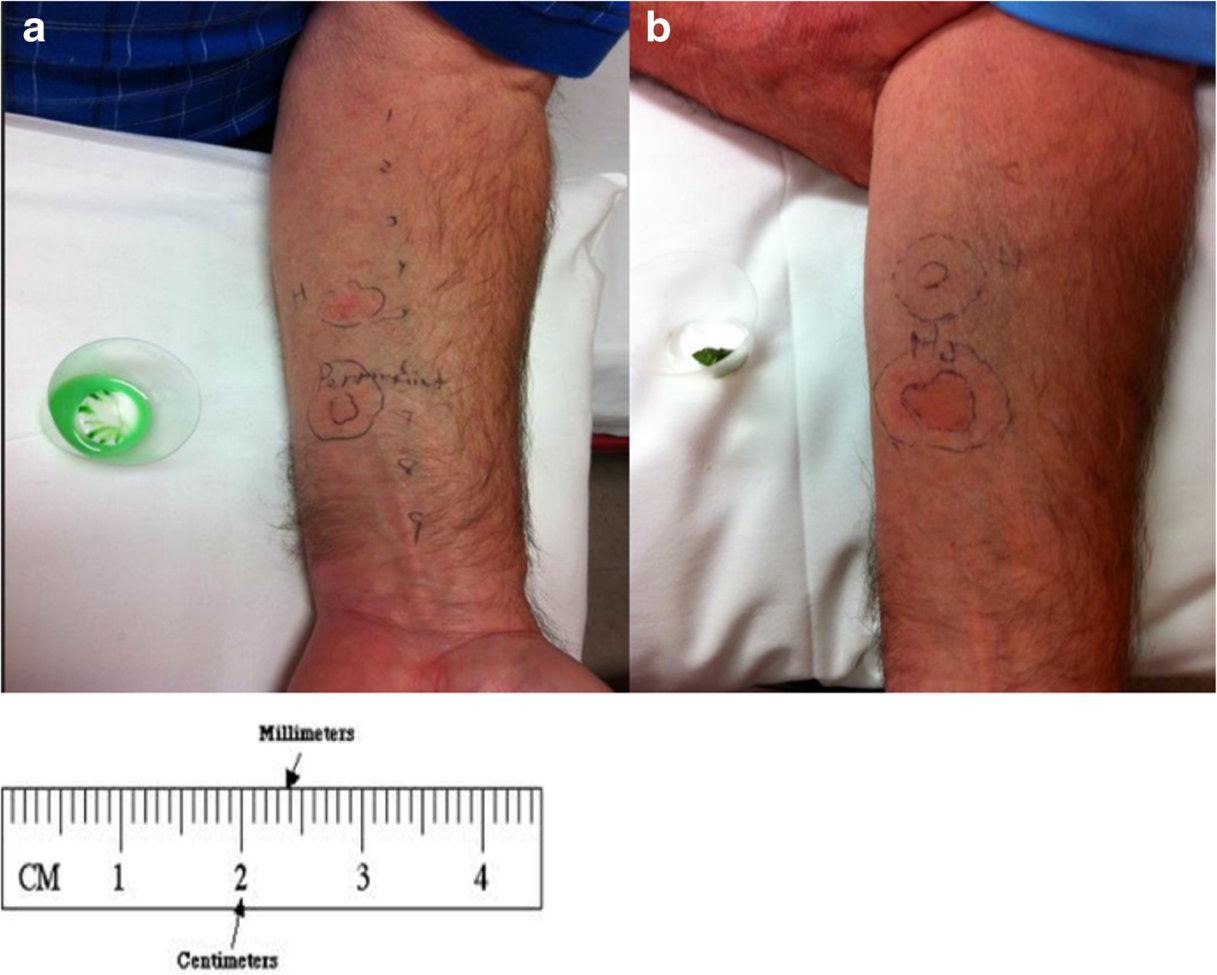
**Skin test responses to peppermint allergen applied on patient’s forearm. a)** Prick test to saline slurry of peppermint candy **b)** Prick-to-prick test to peppermint leaf.

A previously healthy 69 year old male was evaluated in Outpatient Allergy Clinic. He reported a history of sudden onset of lip and tongue swelling, throat tightness, shortness of breath, cough and wheeze within five minutes of sucking on a peppermint candy. He took two tablets of diphenhydramine 25 mg and his symptoms progressed to difficulty with talking and swallowing. He was urgently transported to Emergency Department where he was administered epinephrine intramuscularly, parenteral antihistamines and steroids. His vitals remained stable throughout the episode and he denied any urticarial lesions or gastrointestinal symptoms. His symptoms resolved within 3–4 hours. On history, he reported frequent consumption of foods with peppermint flavour such as chocolates, candies, ice cream, as well as peppermint tooth paste, which he had tolerated well. However, a few weeks prior to this episode, he had developed isolated tongue and lip swelling with the use of Colgate peppermint toothpaste and had avoided it since, although he did not consider the peppermint flavour to be the culprit. During summer months of the preceding three years, he had cultivated peppermint plants in his backyard and admitted to having experienced nasal congestion, cough and a slight wheeze when gardening. His past medical history was remarkable for gout, dyslipidemia and gastroesophageal reflux. He had no prior history of atopy. His medications were simvastatin, omeprazole and aspirin. Skin prick testing was performed to common inhalants and was positive to dust mites, but negative to other common inhalants, including eight types of moulds. Skin testing was negative to commercially available food allergens of egg white, cow’s milk, fish, shellfish, peanut, tree nuts, wheat, soy and sesame. Skin prick testing to a saline slurry of peppermint candy demonstrated a wheal and flare with largest diameters of 10 mm and 35 mm (W10/F25), respectively (Figure 1a). Prick-to-prick test with fresh peppermint leaf provided by the patient revealed a skin test response of W25/F50 (Figure 1b). Both peppermint tests were negative when applied on five healthy volunteers. Patient and healthy volunteers displayed appropriate histamine (W3F25) and negative control (W0F0) skin responses. Our patient was advised to avoid any traces of peppermint and menthol and carry an epinephrine autoinjector. He removed the peppermint plants from his backyard and returned to gardening the following summer without experiencing any symptoms. Later on, he had an accidental exposure to lip balm with traces of peppermint flavour causing solitary lip swelling that responded to oral diphenhydramine.

## Discussion

We describe the case of a 69 y male, who experienced systemic anaphylaxis after sucking on a peppermint candy and have reviewed the relevant literature. Mint plants are members of the genus Mentha, Lamiaceae family which also includes other aromatic plants such as thyme, oregano, basil and rosemary. In Ontario, outdoor mint plants pollinate in July-August period. Menthol (2-isopropyl-5-methyl cyclohexanol) is the naturally occurring active ingredient of peppermint and is widely used because of its distinctive flavour. Allergic contact dermatitis caused by Type IV sensitivity to mint has been well described; however first report of menthol induced allergy was published in 1964 [3]. A 31 y female presented with a 4 day history of generalized urticaria which ceased immediately after stopping all products containing menthol and the sensitization was confirmed by positive immediate contact test to peppermint oil. Case reports of rhinitis and asthma symptoms caused by exposure to peppermint toothpaste and losenges have been previously published [4-7]. The diagnosis of peppermint reaction was confirmed via challenge test with peppermint toothpaste leading to rhinitis symptoms, wheezing and a drop in FEV1. Similarly, our patient experienced upper and lower respiratory symptoms when inhaling peppermint pollen from his garden in the three consecutive summers preceding his anaphylactic reaction.

Paiva et al. published a case of mint toothpaste-induced anaphylaxis in a 46 y female who experienced three anaphylactic reactions after the use of two different brands of toothpaste containing menthol [7]. Subsequently, skin prick test and provocation challenge with a menthol toothpaste were complicated with rhinoconjuctivitis symptoms, abdominal cramps and wheezing, suggesting multisystem involvement similar to our case report.

In most reports published so far, sensitization to peppermint occurred via oral and/or dermal route [3-8]. In contrast, our patient displayed a unique mode of sensitization to peppermint plant, via airborne peppermint pollen [9]. Although he had intermittently consumed foods containing mint flavour all his life and tolerated them well, we believe that inhalation of peppermint pollen when gardening caused sensitization to peppermint which was followed by gradually worsening allergic clinical response when consuming items via oral and contact routes, such as with peppermint toothpaste, candy and lip balm .

Our patient’s IgE mediated sensitization was confirmed by strong positive responses via skin prick test to peppermint candy as well as fresh peppermint leaf from his garden. The same tests were negative in five healthy volunteers verifying that the wheal- and-flare responses noted with the patient were not due to direct mast cell histamine release caused by peppermint, but likely a specific IgE mediated response. Recently, Damiani et al. have published a case report of peppermint induced uvular edema confirmed by positive skin testing to fresh peppermint and also identified a 50-kDa protein to which the patient produced sIgE [10]. Although they conclude that more studies would be needed to confirm these results, it is highly suggestive that peppermint is capable of triggering a specific IgE mediated response.

## Conclusion

This case represents evidence of IgE mediated systemic anaphylaxis to peppermint candy preceded by unique airborne sensitization to peppermint pollen. Peppermint flavour is widely used and our case indicates the future need to consider this allergen as a potential trigger when working up anaphylaxis.

## Consent

Written informed consent was obtained from the patient for the publication of this report and any accompanying images.

## Competing interests

The authors declare that they have no competing interests.

## Authors’ contributions

RB assessed the patient in clinic and RBM supervised the assessment and skin testing performed on the patient and healthy volunteers. RB drafted the abstract, background and parts of clinical history. RBM drafted parts of clinical history, discussion and conclusion. Both authors were involved in revising the manuscript for important intellectual content, and read and approved the final manuscript.
